# Core Genome Multilocus Sequence Typing Scheme for Stable, Comparative Analyses of Campylobacter jejuni and C. coli Human Disease Isolates

**DOI:** 10.1128/JCM.00080-17

**Published:** 2017-06-23

**Authors:** Alison J. Cody, James E. Bray, Keith A. Jolley, Noel D. McCarthy, Martin C. J. Maiden

**Affiliations:** a Department of Zoology, University of Oxford, Oxford, United Kingdom; b NIHR Health Protection Research Unit in Gastrointestinal Infections, University of Oxford, Oxford, United Kingdom; c Warwick Medical School, University of Warwick, Coventry, United Kingdom; University of Iowa College of Medicine

**Keywords:** Campylobacter, molecular epidemiology, whole-genome sequencing, core genome

## Abstract

Human campylobacteriosis, caused by Campylobacter jejuni and C. coli, remains a leading cause of bacterial gastroenteritis in many countries, but the epidemiology of campylobacteriosis outbreaks remains poorly defined, largely due to limitations in the resolution and comparability of isolate characterization methods. Whole-genome sequencing (WGS) data enable the improvement of sequence-based typing approaches, such as multilocus sequence typing (MLST), by substantially increasing the number of loci examined. A core genome MLST (cgMLST) scheme defines a comprehensive set of those loci present in most members of a bacterial group, balancing very high resolution with comparability across the diversity of the group. Here we propose a set of 1,343 loci as a human campylobacteriosis cgMLST scheme (v1.0), the allelic profiles of which can be assigned to core genome sequence types. The 1,343 loci chosen were a subset of the 1,643 loci identified in the reannotation of the genome sequence of C. jejuni isolate NCTC 11168, chosen as being present in >95% of draft genomes of 2,472 representative United Kingdom campylobacteriosis isolates, comprising 2,207 (89.3%) C. jejuni isolates and 265 (10.7%) C. coli isolates. Validation of the cgMLST scheme was undertaken with 1,478 further high-quality draft genomes, containing 150 or fewer contiguous sequences, from disease isolate collections: 99.5% of these isolates contained ≥95% of the 1,343 cgMLST loci. In addition to the rapid and effective high-resolution analysis of large numbers of diverse isolates, the cgMLST scheme enabled the efficient identification of very closely related isolates from a well-defined single-source campylobacteriosis outbreak.

## INTRODUCTION

Campylobacteriosis is a predominant bacterial cause of acute gastroenteritis worldwide, causing substantial morbidity and costs to health care systems, in high-, middle-, and low-income countries (1). In high-income countries such as the United Kingdom (UK) and the United States, the majority (90%) of human disease is caused by Campylobacter jejuni, with Campylobacter coli responsible for most of the remaining cases (2). Both of these organisms are ubiquitously present in the intestines of wild and domesticated animals, where they are thought to be harmless commensal members of the microbiota. They are found at particularly high prevalence in commercial broiler chickens, and there is some evidence that these infections may also be pathological (3). Although C. jejuni and C. coli differ by ∼15% at the nucleotide sequence level across the genome (4), a single multilocus sequence typing (MLST) scheme has been widely adopted for the epidemiological and population analysis of both organisms (5, 6). Given their shared hosts and similar pathologies, the use of a common typing scheme is important for their analysis, and the Campylobacter MLST scheme has been highly successful in elucidating the epidemiology, population structure (7), and evolution (8) of these bacteria. MLST data have also been widely applied in attribution studies, which have implicated contaminated poultry meat as a predominant source of human Campylobacter infection in several settings (9–11).

Single-source outbreaks of campylobacteriosis are considered rare, being associated with the ingestion of raw or incompletely pasteurized milk (12, 13), untreated water (14, 15), and high-risk products such as chicken liver paté (16–18). Over ninety percent of reported human disease is thought to be due to sporadic infection; however, many of these cases may represent diffuse outbreaks. To date, documented continuous-source outbreaks have been associated with contaminated water (19, 20), but many more such outbreaks may occur across wide geographic areas and longer time periods, as a consequence of the consumption of widely distributed foodstuffs. Such outbreaks will be difficult to detect without large-scale surveillance involving high-resolution typing approaches.

The advent of whole-genome sequencing (WGS) technologies for clinical microbiology application (21) has greatly increased the volume of genetic information available for the characterization of bacterial isolates, with simultaneous reductions in cost (22). This has the potential to improve surveillance by the introduction of cost-effective, high-resolution typing systems. Whereas previous typing systems relied on choosing those components of the organism or parts of its genome that were amenable to analysis, WGS enables any part of the genome to be considered as a typing target. The challenges are, therefore, the design, validation, acceptance, and adoption of unified agreed typing schemes (23) from the plethora of those that can be envisaged. For Campylobacter, universal single- and multiple-locus typing schemes, including antigen gene typing (24), conventional seven-locus MLST (5), and ribosomal sequence typing (rMLST) (25, 26), have been designed and implemented with internationally accepted Web-based nomenclature servers available (27), but none of these has the resolution to identify diffuse outbreaks or diversity within outbreaks.

Here we present a set of loci for use as a core genome MLST (cgMLST) scheme for C. jejuni and C. coli in the analysis of human campylobacteriosis isolates. The scheme has been validated against a large number of representative isolates from the UK, Europe, and North America and is versioned (version 1.0 is described here) to enable consistent analyses to be performed among different laboratories in different jurisdictions without the necessity of sharing isolates or data.

## RESULTS

**FIG 1:**
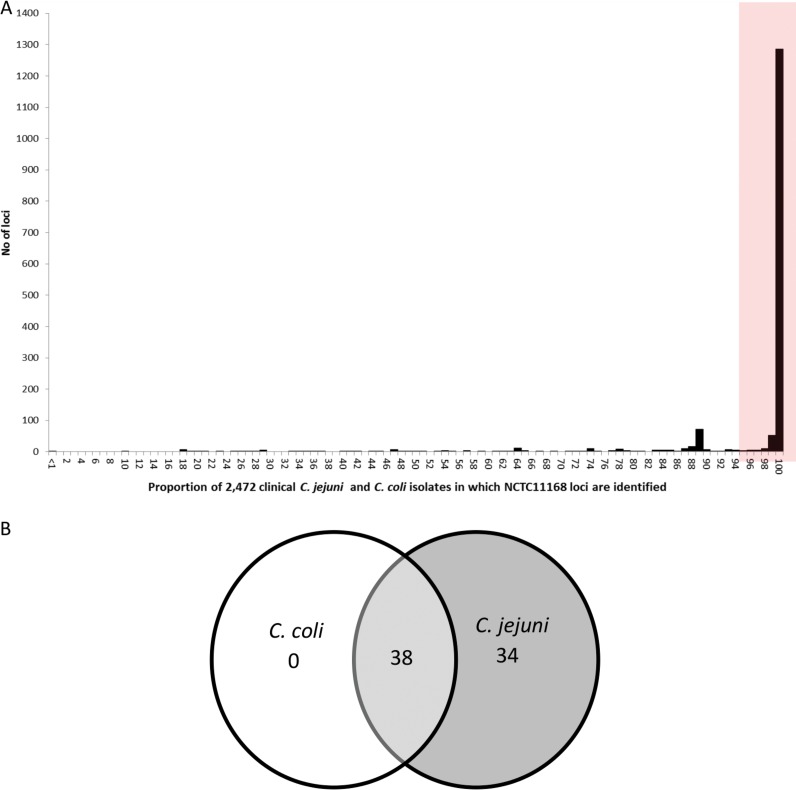
(A) Numbers of 1,643 loci defined in the reannotation of reference genome NCTC 11168, identified in 2,472 clinical C. jejuni (2,207) and C. coli (265) isolates, from Oxfordshire, UK. The area under the shaded box includes 1,365 loci identified in 95% or more of the isolates from both species. (B) Reanalysis of the 72 loci identified under the peak at 89% in panel A, indicating the number of loci identified in genomes from C. coli only or C. jejuni only or that are present in both species.

Of the 1,643 coding sequences identified in C. jejuni reference strain NCTC 11168, 1,365 were found to be present in 95% or more of the 2,472 Oxfordshire clinical draft genomes (Fig. 1A). A secondary minor peak, indicating that an additional 72 loci were identified in only 89% of the isolates, was observed, and reanalysis to establish the presence of these loci in isolates belonging to C. jejuni or C. coli determined that these loci were predominantly present only in C. jejuni isolates (Fig. 1B). A total of 22 potential paralogous loci (Table 1) were identified and removed from the original list of 1,365 core genes, to give a cgMLST scheme of 1,343 loci, available at http://pubmlst.org/campylobacter.

**TABLE 1:** Twenty-two potential paralogues removed from the initial C. jejuni/C. coli cgMLST v1.0 scheme of 1,365 loci

Gene designation	CAMP no.	Gene product
Cj0045c	CAMP0044	Putative iron-binding protein
Cj0072c	CAMP1625	Pseudogene (putative iron-binding protein)
Cj0251c	CAMP0221	Highly acidic protein
Cj0416	CAMP0381	Hypothetical protein
Cj0770c	CAMP0705	Putative NLPA family lipoprotein
Cj0771c	CAMP0706	Putative NLPA family lipoprotein
Cj0772c	CAMP0707	Putative NLPA family lipoprotein
Cj0814	CAMP0749	Hypothetical protein
Cj0816	CAMP0751	Hypothetical protein
Cj0851c	CAMP0786	Putative integral membrane protein
Cj1018c/livK	CAMP0941	Branched-chain amino acid ABC transport system, periplasmic binding protein
Cj1019c/livJ	CAMP0942	Branched-chain amino acid ABC transport system, periplasmic binding protein
Cj1149c/gmhA	CAMP1068	Sedoheptulose 7-phosphate isomerase
Cj1189c/cetB	CAMP1108	Bipartate energy taxis response protein CetB
Cj1191c	CAMP1110	Putative PAS domain containing signal transduction sensor protein
Cj1200	CAMP1119	Putative NLPA family lipoprotein
Cj1224	CAMP1143	Putative iron-binding protein
Cj1305c	CAMP1223	Hypothetical protein
Cj1306c	CAMP1224	Hypothetical protein
Cj1310c	CAMP1228	Hypothetical protein
Cj1342c/maf7	CAMP1258	Motility accessory factor
Cj1360c	CAMP1276	Putative proteolysis tag for 10Sa_RNA

Of the 19 pseudogenes identified in the reannotation of the reference strain NCTC 11168 (28), only one (Cj0072c) of the candidate loci for inclusion in the cgMLST scheme was also identified as a paralogue and therefore excluded. The 1,343 core loci included only seven potential pseudogenes, which were retained in the scheme because (i) between 36.9% and 100% of alleles at these loci in the 2,472 Oxfordshire clinical isolate samples set as of 16 November 2016, and which did not include the reference strain, represented coding sequence (Table 2) and (ii) no other loci were excluded from the scheme on the grounds that one or more alleles were noncoding in any of the genomes.

**TABLE 2:** Pseudogenes identified in the reference genome and numbers of coding and noncoding allele sequences identified in the 2,472 clinical isolates from Oxfordshire, United Kingdom

Gene designation	CAMP no.	No. (%) of coding alleles	No. (%) of noncoding alleles	Total no. of alleles
Cj0292c	CAMP1638	64 (82.1)	14 (17.9)	78
Cj0444	CAMP1627	98 (39.8)	148 (60.2)	246
Cj1064	CAMP1637	164 (100)	0 (0.0)	164
Cj1389	CAMP1639	87 (36.9)	149 (63.1)	236
Cj1395	CAMP1640	151 (69.9)	65 (30.1)	216
Cj1470c	CAMP1641	129 (91.5)	12 (8.5)	141
Cj1528	CAMP1642	57 (78.1)	16 (21.9)	73

Putative functions were assigned to 1,301 (96.9%) of the 1,343 core loci, using the RAST server (29), with 25 functional categories represented by between 1 (0.1%) and 257 (19.8%) genes. The highest proportion of genes were associated with the metabolism of amino acids and derivatives (19.8%), proteins (15.4%), and cofactors, vitamins, prosthetic groups, and pigments (10.6%) (see Table S1 in the supplemental material). The remaining 23 categories were represented by 5.4% of loci or fewer.

**FIG 2:**
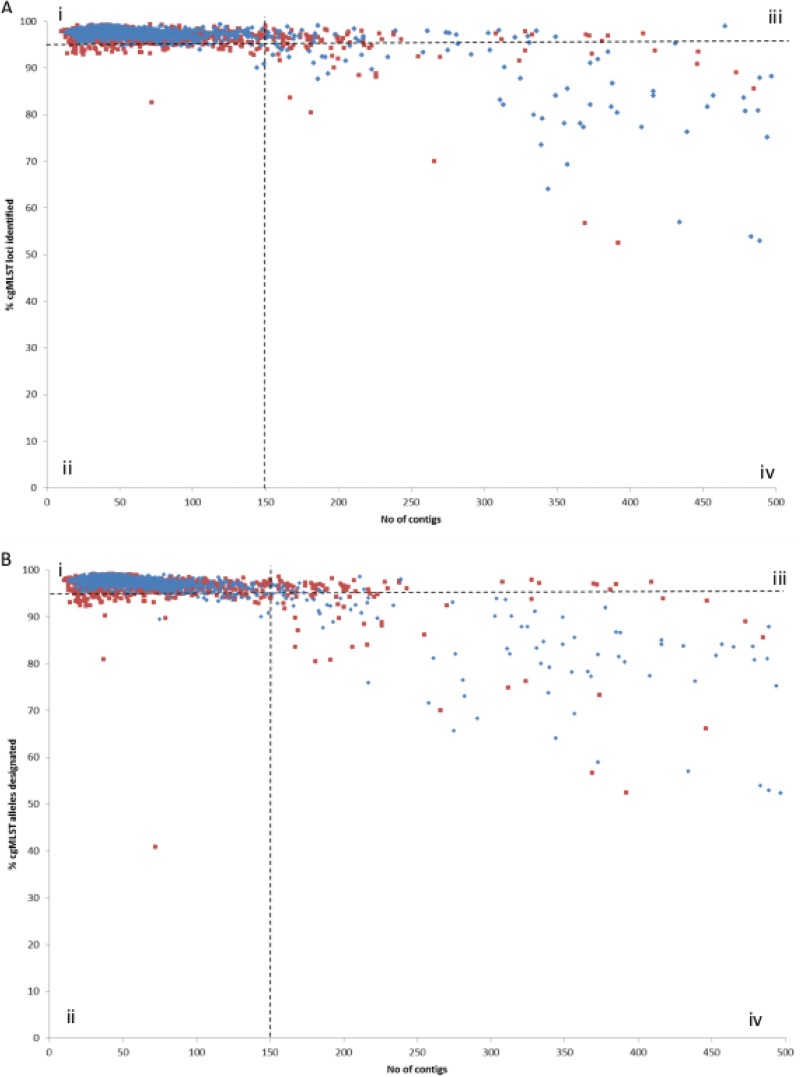
Validation of the cgMLST scheme by assessment of the proportion of clinical (blue) and nonclinical (red) isolates in which the core loci and defined allele sequences could be identified. (A) Percentage of 1,343 cgMLST loci identified; (B) proportion of these loci from panel A with an allele designated in 2,945 C. jejuni and C. coli isolates. Broken lines indicate the cutoff values for isolates in which 95% of loci were identified or alleles designated and the numbers of contigs from which these data could be reliably informed. Quadrants thus defined are labeled as i, ii, iii, and iv, respectively, with details of these for each isolate detailed in Table S5.

The scheme was validated by assessment of the proportion of cgMLST loci detected and alleles identified in isolate collections from Europe and North America of (i) 1,574 clinical isolates (1,349 C. jejuni and 225 C. coli isolates) and (ii) 1,371 animal and environmental genomes (from 781 C. jejuni and 653 C. coli isolates) available from the PubMLST database (Fig. 2; see also Tables S3 and S5 in the supplemental material). Ninety-five percent or more of the 1,343 cgMLST loci were present in 1,510 (95.9%) isolates from this clinical isolate collection (Fig. 2A), with an association between the number of cgMLST loci identified and the number of contigs. Of the 1,478 clinical genomes that comprised 150 contigs or fewer, only seven isolates (0.5%) had the positions of <95% loci identified (tagged). After a BLAST search against the cgMLST sequence definition database allele library, 1,452 of these 1,478 (98.2%) genomes were found to have 95% or more of the cgMLST alleles designated (Fig. 2B). When this analysis was applied to the 1,371 genomes from animal and environmental sources, 95% or more of the cgMLST loci were detected in 1,279 (93.3%) isolates (Fig. 2A), of which 1,252 (91.3%) had 95% or more of cgMLST alleles designated (Fig. 2B). For the 1,278 (93.2%) nonclinical genomes with 150 contigs or fewer, 1,222 (95.6%) had 95% or more cgMLST loci identified, and 1,200 (93.9%) of these had 95% or more of the cgMLST alleles designated.

**FIG 3:**
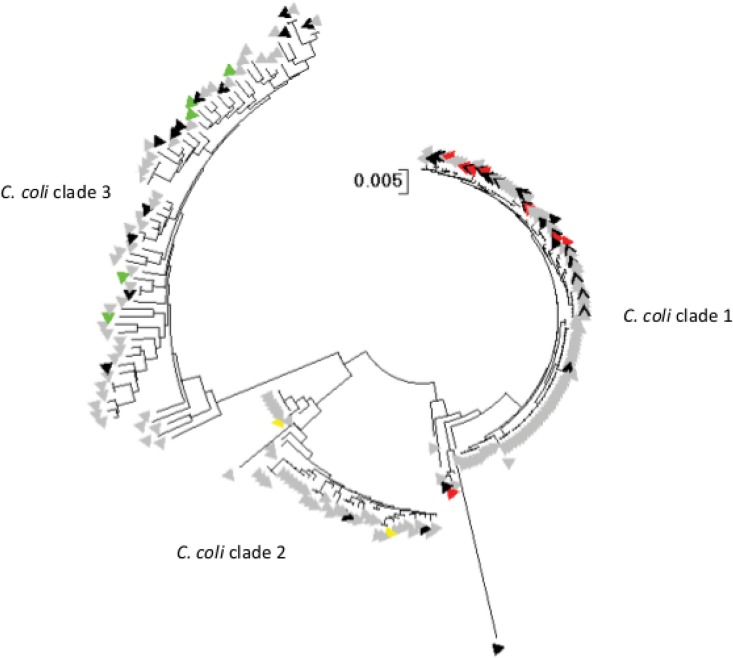
Identification of C. coli genomes unassigned to a clonal complex from clinical (*n* = 42) (black triangles) and nonclinical (*n* = 175) (gray triangles) isolates belonging to clades 1, 2, and 3. Concatenated sequences from seven-locus MLST alleles of isolates used to validate the human disease cgMLST scheme v1.0 were used to construct a neighbor-joining tree, which included reference isolates of known C. coli clades that are colored as follows: red, C. coli clade 1; yellow, C. coli clade 2; green, C. coli clade 3. Reference isolates are detailed in Table S2. The scale bar represents the *p*-distance between aligned sequences.

There are three C. coli clades described to date, using phylogenetic analysis of 7-locus MLST, which are associated with different host sources (8). Clade 1 C. coli, largely represented by isolates belonging to ST-828 and ST-1150 clonal complexes (cc), are most commonly associated with human campylobacteriosis and food animals, whereas C. coli clades 2 and 3 are more usually isolated from wild birds and environmental sources. Although the cgMLST scheme was established from clinical genomes, it was important to assess the extent to which variation among clade 2 and clade 3 C. coli isolates was identified. Of the 265 C. coli isolates in the 2,472 Oxfordshire clinical isolate data set used to determine the core genome, 23 were not members of ST-828 cc or ST-1150 cc and therefore did not belong to C. coli clade 1. Comparison of these genomes with those of C. coli isolates of known clade assignment (see Table S2 in the supplemental material) by phylogenetic analysis identified three genomes as belonging to clade 3 (data not shown). When clinical isolates used for validation of the cgMLST scheme (*n* = 42) that were unassigned to either of these clonal complexes were compared to the reference genomes, by phylogenetic analysis of data from the MLST scheme, 25 were identified as belonging to clade 1, two isolates to clade 2, and 15 isolates to clade 3 (Fig. 3). Of these, 20 (80.0%), 1 (50.0%), and 10 (62.5%) isolates, respectively, were represented by 150 contigs or fewer, from which 95% or more loci were tagged; however, two clade 1 genomes and one clade 2 genome with 150 contigs or fewer had less than 95% of alleles designated. A comparison of nonclinical (*n* = 175) unassigned C. coli isolates used for scheme validation, with the same reference genomes, determined the presence of 80 clade 1, 41 clade 2, and 54 clade 3 isolates (Fig. 3), 70 (83.8%), 37 (90.2%), and 22 (40.7%) of which, respectively, had genomes comprised of 150 contigs or fewer, in which 95% or more of loci were tagged and 95% or more of cgMLST alleles were designated; fewer than 95% of alleles were designated in six clade 3 genomes comprising fewer than 150 contigs. A single isolate (id 31118), confirmed as C. coli by rMLST, did not have a complete MLST profile and was excluded from further analysis.

**FIG 4:**
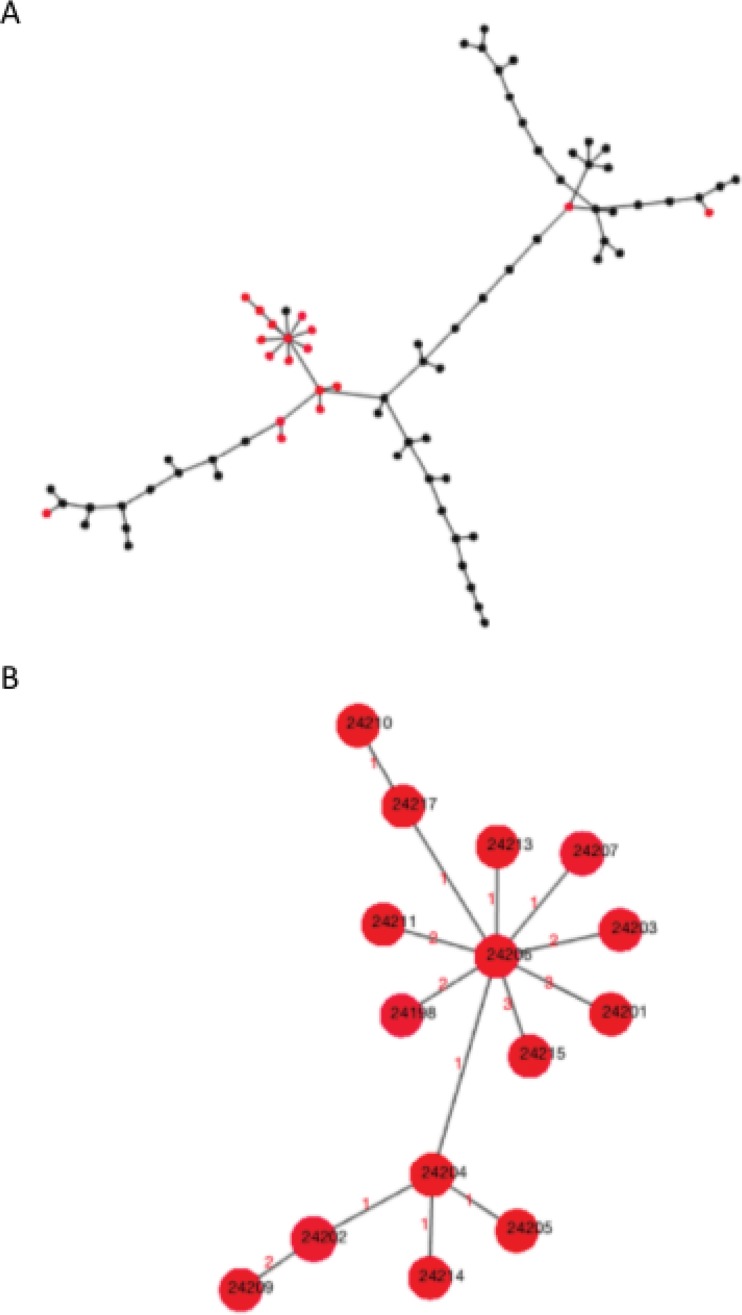
(A) Single-linkage cluster analysis of cgMLST allelic profiles visualized as a minimum spanning tree in PHYLOViZ, from 23 potential outbreak isolates (red) and 59 contemporaneous surveillance isolates from Oxfordshire, UK (black). (B) Twenty clustered outbreak isolates represented by 15 cgMLST profiles, as represented by a single isolate identifier, indicated in black type. The numbers of allelic differences between cgMLST profiles are indicated in red. Link lengths are not proportional to the number of allelic differences.

The comparison of cgMLST allelic profiles from a previously published data set of 23 isolates associated with a known outbreak and 59 contemporaneous Oxfordshire surveillance C. jejuni samples (13), visualized as a minimum spanning tree in PHYLOViZ, identified a cluster of 15 allelic profiles representing 20 outbreak isolates (Fig. 4). Within this cluster, differences between isolates predominantly arose in instances where loci occurred on the end of a contig, from which complete locus detection and allele designations could not be made. Three potential outbreak-associated genomes were genetically distant from the cluster of 20 but were highly similar to contemporaneous isolates, with one differing by only 3 alleles from the genome of a control isolate from Oxfordshire. Phylogenetic comparison of the concatenated cgMLST nucleotide sequences identified the same isolate clusters as those obtained with allelic profiles (see Fig. S1 in the supplemental material).

## DISCUSSION

Genomic studies of multiple bacterial isolates have enabled the establishment of the concepts of (i) the core genome, i.e., those genes present in most or all bacterial isolates in a particular group, and (ii) the whole genome, the complete genetic complement of a constrained number of related isolates, as defined by the full complement of loci from a defined reference genome. In practice, very high resolution can be attained across large groups of isolates by core genome comparisons, which have the advantage of being highly reproducible across data sets, although increased resolution can be achieved using whole-genome analysis. These high-resolution MLST-like approaches to the genomic comparison of bacterial isolates can be systematized as core genome MLST (cgMLST) and whole-genome MLST (wgMLST) (23, 26).

Various definitions can be used to identify the members of a core genome (23). The most stringent definition, designating as part of the core genome those genes that are present in all isolates, is problematic, first, because all isolates are potential mutants, resulting in a progressive reduction in core genome size as more isolates are examined, and second, because the WGS approaches used at the time of writing result in draft, incomplete genome sequences in which some genes may be missing (30). A number of approaches have been used to define core genomes in Campylobacter, leading to estimated core genome sizes of 847 (31), 866 (32), 1,001 (33), 1,035 (34), and 1,295 (35) loci. The number of loci in the core genome set varies depending on the algorithms used and the cutoff values employed, combined with the source, species, and genomic diversity of the isolates examined. Here we undertook a survey of large collections of WGS data of human campylobacteriosis isolates to identify the maximum number of genes for use in a cgMLST scheme. The aim was to propose a set of genes that can be used as a basis for reproducible comparisons among clinical laboratories interested in human campylobacteriosis.

The reannotated genome sequence of the reference isolate NCTC 11168 (36) was used as the primary source for the 1,643 candidate protein-encoding loci to be included in the core genome scheme (28). A set of 1,343 genes with single loci (82% of the genome) was generated by using a 95% threshold for the presence of loci in the 2,472 human campylobacteriosis isolates (Fig. 1), followed by the removal of paralogous genes, a potential source of inaccuracies in gene-by-gene comparisons. These 1,343 loci were included in the human campylobacteriosis cgMLST scheme, version 1.0, in which the proportion of genes belonging to different functional categories were largely comparable to that of the 1,643 loci from the NCTC 11168 genome (Table S1) (28). This scheme, including the allele sequences for each of the loci included, is available from https://pubmlst.org/campylobacter/, where it can be accessed directly or through a RESTful API (http://rest.pubmlst.org) of the BIGSdb database (37). The version number for the scheme means that it is possible to make refinements while retaining the possibility of using previous versions for comparative analyses or to reproduce work published with the scheme. Any such changes would affect only the complement of genes included and not allele designations made at each locus.

Using a single reference genome as a source of loci potentially results in loci that are present in the majority of isolates but are not present as a functional gene in the reference isolate, being excluded from the cgMLST scheme. This is especially a concern in Campylobacter, in which organism a large number of genes are potentially phase variable and present as nonfunctional genes in some isolates (36). Nineteen such genes are identified in the reannotation of NCTC 11168 (28). These genes were not removed from this analysis, and after the removal of potential paralogues, seven of them were included in cgMLST v1.0. Interestingly, only one of these (Cj1064, PubMLST locus CAMP1637) was “phase variable off” in NCTC 11168 and “phase variable on” in all members of the reference set of human campylobacteriosis isolates (Table 2). The remaining phase-variable genes were phase variable on in 36.9 to 100.0% of the alleles identified in the human campylobacteriosis isolates (Table 2). Thus, it is unlikely that many functional genes have been excluded from cgMLST v1.0 due to the use of the single reference genome.

One of the challenges in the application of WGS approaches to clinical and public health problems is the incompleteness of the data that can be available from clinical specimens. This is exacerbated by the use of draft sequences, which may be in multiple contiguous sequences (contigs) and may have many loci missing (30). In both the clinical and nonclinical data sets analyzed here, 95% of the cgMLST loci were detected in the majority (97.6%) of those genome assemblies with 150 contigs or fewer, which can be used as a quality threshold for the analysis of such data. It was noteworthy that for some isolates with substantially more than 150 contigs, it was still possible to detect more than 95% of the cgMLST v1.0 genes.

There are three known clades of C. coli (referred to as clades 1, 2, and 3) (8), with clade 1 being the most commonly associated with human disease and clades 2 and 3 more commonly present among isolates from wild birds and environmental sources. Clade 1 C. coli isolates show the most evidence for introgression, i.e., gene acquisition, from C. jejuni (4). As expected, there were a lower proportion of the cgMLST v1.0 genes detected in C. coli clade 2 and clade 3 isolates, present in the second set of isolates examined. Although cgMLST v1.0 will provide some resolution of such isolates, it was formulated for the analysis of human campylobacteriosis isolates and this is its recommended use. For detailed analysis of C. coli as a species, an alternative cgMLST scheme should be developed. The analysis presented here suggests that a cgMLST scheme for C. jejuni alone would be 70 to 80 loci larger (Fig. 1B), which is unlikely to substantially improve resolution over the one that we propose, covering both species.

The reanalysis of a previously published human campylobacteriosis outbreak (13) with cgMLST v1.0 demonstrated the comparability of the results obtained and the ease with which results of cgMLST analyses can be manipulated. Within a group of 23 potential outbreak isolates, 20 were found to represent a single strain, indicating that they most likely shared a common point source. When compared with isolates concomitantly sampled in a geographically distant surveillance area, one of the three isolates disparate from the outbreak strain was found to differ at only three loci from a surveillance isolate. This finding adds further support to the hypothesis that many disease isolates may represent continuous source outbreaks, acquired via extended food distribution networks. The cgMLST allelic profile comparisons were directly comparable with the originally reported findings, obtained using wgMLST (13), and with those from phylogenetic analysis of concatenated cgMLST allele sequences (Fig. S1).

In conclusion, the cgMLST v1.0 genes set proposed here provides a high-resolution WGS analysis scheme for isolates from human campylobacteriosis, which can be used both for ongoing disease surveillance and the resolution of very closely related isolates obtained during outbreak investigation. The ability to group isolates using this scheme provides, for the first time, an automated means of detecting clusters from a diffuse set of isolates rapidly using Web-based tools. The scheme is freely available via the PubMLST.org database and in machine-readable format via the RESTful API, enabling its incorporation into other analysis platforms, as required.

## MATERIALS AND METHODS

### Core gene identification.

Whole-genome sequence data from 2,472 clinical C. jejuni (*n* = 2,207) and C. coli (*n* = 265) isolates, each representing a unique infection and with complete MLST and rMLST profiles, from Oxfordshire, UK, between 2011 and 2014, were obtained as previously described (26). Contiguous sequences for each isolate were scanned by BIGSdb software (37), and the positions of the 1,643 loci were recorded (“tagged”) in each draft genome. Coding sequence was identified by alleles with in-frame start and stop codons; these were initially indicated by the reference allele from NCTC 11168, but as the number of genomes increased, some start codons that were not necessarily in accordance with that defined in the initial reference were identified, and codons were then identified as dictated by the remainder of the data set. Alleles without in-frame start and/or stop codons were regarded as noncoding, and their assigned alleles were flagged as such.

The annotated reference strain used to seed the database was representative of the most abundant, multihost clonal complex (ST-21 complex) causing human disease in the UK (2, 26). The presence of these loci in each draft genome was compared using BLASTN to identify genes with ≥70% sequence identity to ≥50% of the length of the locus. Loci found to be absent in no more than 5% of isolates (i.e., those present in ≥95.0% of isolates) were included within the scheme. This cutoff level was chosen (i) to take into account the draft nature of the genomes, in which all regions may not sequence or assemble completely, and (ii) to prevent the exclusion of loci that encode conserved essential functions but are inactive in particular rare isolates.

### Paralogue and pseudogene identification.

**FIG 5:**
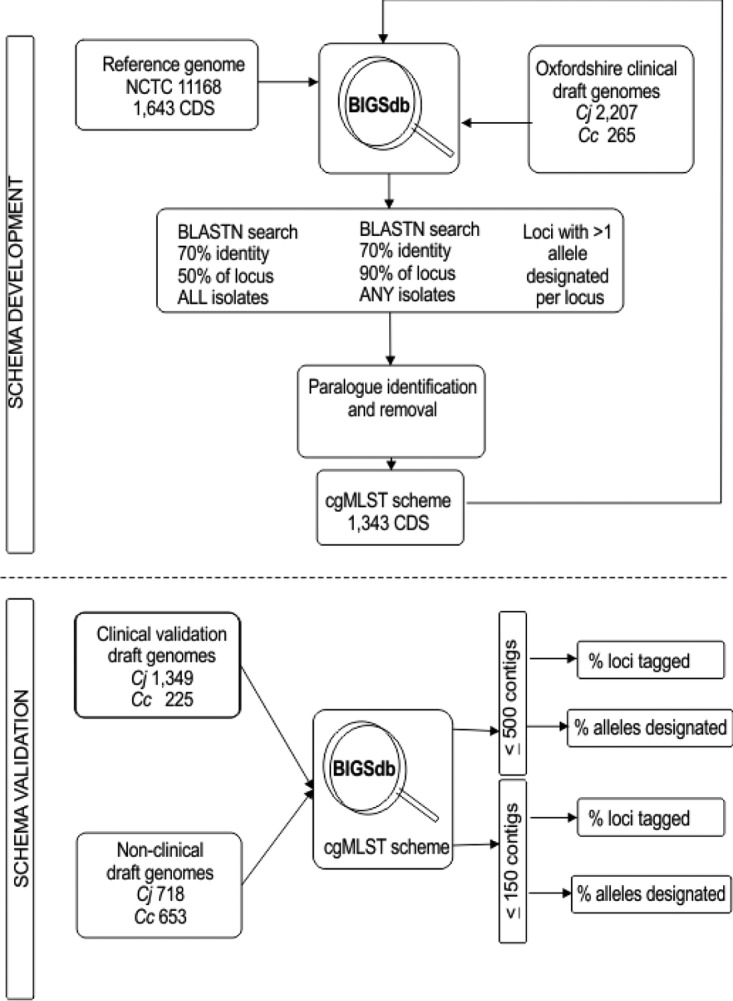
Diagrammatic representation of the basic methodology used for (i) identification of cgMLST genes from Oxfordshire clinical draft genomes and paralogous loci, for the development of the cgMLST scheme (top), and (ii) validation of the scheme using clinical and nonclinical draft genomes available in pubmlst.org/campylobacter (bottom).

Inaccuracies may arise in core genome comparisons when two different genes are so similar that their alleles can be assigned to multiple loci in the genome. To prevent such errors, potential paralogous loci were excluded from the core genome definition by their identification from five subsets of 10 isolates (Table S4), chosen to represent the diversity of clonal complexes causing the majority of human disease, using a variety of methods which are as follows (Fig. 5). The 50 genomes were compared using the genome comparator function of BIGSdb to identify paralogous loci for exclusion from the cgMLST scheme as follows: (i) those loci that were paralogous in all 50 isolates, using the BLAST settings detailed above, and (ii) those loci that were paralogous in any of the 50 isolates, but with the requirement for ≥70% sequence identity to a minimum of 90% of the locus. Additionally, the 2,472 draft genomes were searched for loci at which more than one allele was designated. The similarities of sequences thus identified to loci present elsewhere in the genome were also investigated. Finally, genes identified by any of these analyses that were also present in the initial 95% core genome were removed from the scheme.

Loci identified as pseudogenes in the reannotation given in reference 28 that were candidate members of the cgMLST scheme were investigated for the presence of coding sequence in the 2,472 genomes, by aligning translated allele sequences contained in pubmlst.org/campylobacter and analysis with MEGA v5.1 (38). The whole-genome sequence of the reference genome and the concatenated allele sequences for cgMLST loci were downloaded from pubmlst.org/campylobacter and submitted to the RAST server (29) for annotation of putative functional categories.

### Validation analyses.

The cgMLST scheme was validated by identification of the 1,343 loci in 1,574 draft clinical C. jejuni (1,349) and C. coli (225) genomes, obtained from Europe and North America, available at pubmlst.org/campylobacter (Tables S3 and S5). These draft genome assemblies were chosen such that each had a total length of 2 Mb or less and fewer than 500 contigs. These criteria were instituted to minimize mixed cultures and poor-quality sequencing. Allele sequences of the cgMLST loci were automatically scanned, sequences were tagged, and alleles were assigned and incorporated into the sequence definition database allele library, using the BIGSdb autotagger facility. In a further validation step, the analysis was extended to include an additional 1,371 (total, 2,945) similarly chosen C. jejuni (718) and C. coli (653) isolates from animal and environmental sources available in the PubMLST database (Table S5).

The extent to which the cgMLST scheme accurately identified variation among genomes obtained from C. coli isolates belonging to clades 1, 2, and 3 was assessed by means of a neighbor-joining tree of seven-locus MLST concatenated-nucleotide data, reconstructed using MEGA 5.1 software (38), and by comparison with reference isolates (see Table S2 in the supplemental material). Clade 1 C. coli isolates are most commonly isolates from agricultural and clinical sources, whereas clades 2 and 3 are more frequently found in riparian environments (4).

The potential of this cgMLST scheme to distinguish potential outbreak isolates was investigated by comparison of 23 genomes obtained from a geographically isolated human population and 59 contemporaneous clinical C. jejuni genomes from Oxfordshire, UK, which had been previously analyzed by seven-locus MLST and wgMLST (13). Core-genome MLST types (cgST) were assigned to allelic profiles that had up to 100 missing alleles. Missing alleles were replaced in the profile by an “N.” A cgST was added to a single-linkage group if it was linked with at least one other member of that group with less than or equal to the threshold number of allelic differences, where the value N matched any other locus. Core genome STs were automatically assigned to single-linkage clusters, comprising isolates that differed at fewer than 5, 10, 25, 50, 100, or 200 cgMLST loci, as implemented in BIGSdb version 1.14.0. These allele-based isolate clusters were visualized in a minimum spanning tree using PHYLOViZ (39) and compared with those observed by phylogenetic analysis of the concatenated allele sequences of the 1,343 core loci.

## Supplementary Material

Supplemental material
