# Supplementary material for: Core Genome Multilocus Sequence Typing Scheme for Stable, Comparative Analyses of Campylobacter jejuni and C. coli Human Disease Isolates
Source: J Clin Microbiol. 2017 Jun 23;55(7):2086–97. doi: 10.1128/JCM.00080-17 (PMC5483910; doi:10.1128/JCM.00080-17)
Supplement: Supplemental material [file supp_55_7_2086__index.html]

Supplemental material 

# Core Genome Multilocus Sequence Typing Scheme for Stable, Comparative Analyses of Campylobacter jejuni and C. coli Human Disease Isolates

## Supplemental material

- Supplemental file 1 -

  Tables S1 (Functional categories of genes from the reference strain NCTC 11168 identified in the complete genome and the human disease cgMLST scheme v1.0, as identified by RAST), S2 (Reference isolates used to identify *Campylobacter coli* belonging to clades 1, 2, and 3), and S4 (Fifty *Campylobacter* isolates chosen to represent the diversity of clonal complexes causing the majority of human disease used for the detection of paralogous genes) and Fig. S1 (Identification of outbreak isolates from a contemporaneous population of clinical samples using cgMLST)

  PDF, 341K
- Supplemental file 2 -

  Table S3 (Clonal complex and sequence types of isolate populations used for development and validation of the *C. jejuni* and *C. coli* human disease cgMLST scheme v1.0)

  XLSX, 36K
- Supplemental file 3 -

  Table S5 (PubMLST identification numbers of isolates used to validate the human disease 1,343-cgMLST-locus scheme)

  XLSX, 84K
