# Supplementary material for: Core Genome Multilocus Sequence Typing Scheme for Stable, Comparative Analyses of Campylobacter jejuni and C. coli Human Disease Isolates
Source: J Clin Microbiol. 2017 Jun 23;55(7):2086–97. doi: 10.1128/JCM.00080-17 (PMC5483910; doi:10.1128/JCM.00080-17)
Supplement: Supplemental material [file JCM.00080-17_zjm999095549s1.pdf]

	NCTC 11168 complete genome		cgMLST loci	
**Functional Category**	**No of genes**	**Proportion of genes**	**No of genes**	**Proportion of genes**
Amino Acids and Derivatives	287	18.7%	257	19.8%
Protein Metabolism	218	14.2%	200	15.4%
Cofactors, Vitamins, Prosthetic Groups, Pigments	133	8.7%	138	10.6%
Cell Wall and Capsule	118	7.7%	60	4.6%
Motility and Chemotaxis	86	5.6%	68	5.2%
Respiration	73	4.7%	70	5.4%
RNA Metabolism	71	4.6%	69	5.3%
Virulence, Disease and Defence	69	4.5%	54	4.2%
Fatty Acids, Lipids and Isoprenoids	64	4.2%	63	4.8%
Carbohydrates	63	4.1%	55	4.2%
Iron Acquisition and Metabolism	53	3.4%	8	0.6%
Membrane Transport	52	3.4%	36	2.8%
DNA Metabolism	52	3.4%	36	2.8%
Nucleosides and Nucleotides	50	3.3%	49	3.8%
Stress Response	43	2.8%	42	3.2%
Cell Division and Cell Cycle	22	1.4%	22	1.7%
Phosphorus Metabolism	20	1.3%	20	1.5%
Regulation and Cell Signalling	17	1.1%	17	1.3%
Potassium Metabolism	15	1.0%	8	0.6%
Nitrogen Metabolism	14	0.9%	12	0.9%
Sulphur Metabolism	5	0.3%	5	0.4%
Miscellaneous	4	0.3%	4	0.3%
Secondary Metabolism	4	0.3%	4	0.3%
Metabolism and Aromatic Compounds	3	0.2%	3	0.2%
Dormancy and Sporulation	1	0.1%	1	0.1%
**Total genes of assigned function**	**1,537**		**1,301**	

Table S1. The functional categories of genes from the reference genome NCTC11168 identified in the complete genome and the human disease core genome MLST scheme v1.0, as identified by RAST.

Pubmlst id	Isolate	Source	Coli clade	ST	Clonal complex	Reference
32298	15-53760	Clinical	1	855	ST-828	Pearson, 2013
11135	Cc317/04	Clinical	1	5160	ST-828	Lefebure, 2010
35247	Cc90-3	Pig	1	3862		Lefebure, 2010
35277	Cc151-9	Pig	1	1102		Lefebure, 2010
16237	OXC6443	Clinical	1	5736	ST-1150	pubmlst.org/campylobacter
23494	OXC5486	Clinical	1	5158	ST-828	pubmlst.org/campylobacter
23502	OXC5605	Clinical	1	832	ST-828	pubmlst.org/campylobacter
23515	OXC5629	Clinical	1	855	ST-828	pubmlst.org/campylobacter
24267	2544	Clinical	2	2326		Sheppard, 2012
24268	6873	Duck	2	2016		Sheppard, 2012
24269	dfvf1656	Chicken offal or meat	2	1572		Sheppard, 2012
24270	FSA05.280042	Environmental waters	3	1992		Sheppard, 2012
24271	8096	Chicken offal or meat	3	2681		Sheppard, 2012
24272	dfvf1912	Chicken offal or meat	3	1576		Sheppard, 2012
24273	4944	Clinical	3	1670		Sheppard, 2012
24274	RM4931	Clinical	3	1643		Sheppard, 2012

Table S2. Reference isolates used to identify *Campylobacter coli* belonging to clades 1, 2 and 3.

PubMLST_id	isolate	ST (MLST)	clonal_complex (MLST)
28684	OXC8492	2314	ST-1034 complex
28643	OXC8452	122	ST-206 complex
28924	OXC8590	122	ST-206 complex
28983	OXC8598	19	ST-21 complex
28922	OXC8586	50	ST-21 complex
28972	OXC8658	50	ST-21 complex
28981	OXC8405	50	ST-21 complex
28931	OXC8599	53	ST-21 complex
28619	OXC8428	861	ST-21 complex
28644	OXC8453	22	ST-22 complex
28620	OXC8429	257	ST-257 complex
28930	OXC8597	257	ST-257 complex
28934	OXC8602	990	ST-257 complex
28679	OXC8486	353	ST-353 complex
28933	OXC8601	353	ST-353 complex
28628	OXC8437	354	ST-354 complex
28945	OXC8618	354	ST-354 complex
28995	OXC8660	354	ST-354 complex
28943	OXC8616	403	ST-403 complex
28994	OXC8659	42	ST-42 complex
28916	OXC8579	447	ST-42 complex
28624	OXC8433	51	ST-443 complex
28990	OXC8625	51	ST-443 complex
28676	OXC8483	45	ST-45 complex
28663	OXC8470	2844	ST-460 complex
28645	OXC8454	5136	ST-464 complex
28657	OXC8464	5136	ST-464 complex
28950	OXC8632	5136	ST-464 complex
28621	OXC8430	48	ST-48 complex
28966	OXC8652	48	ST-48 complex
28929	OXC8596	775	ST-52 complex
28917	OXC8580	574	ST-574 complex
28979	OXC8572	574	ST-574 complex
28730	OXC8540	1040	ST-574 complex
28926	OXC8593	607	ST-607 complex
28637	OXC8446	61	ST-61 complex
28958	OXC8643	61	ST-61 complex
28651	OXC8460	312	ST-658 complex
28982	OXC8530	658	ST-658 complex
28918	OXC8581	1044	ST-658 complex
28923	OXC8587	1044	ST-658 complex
28712	OXC8521	825	ST-828 complex
28962	OXC8648	825	ST-828 complex
28625	OXC8434	827	ST-828 complex
29001	OXC8667	860	ST-828 complex
28949	OXC8631	881	unassigned

Table S4. Fifty *Campylobacter* isolates chosen to represent the diversity of clonal complexes causing the majority of human disease used for the detection of paralogous genes.

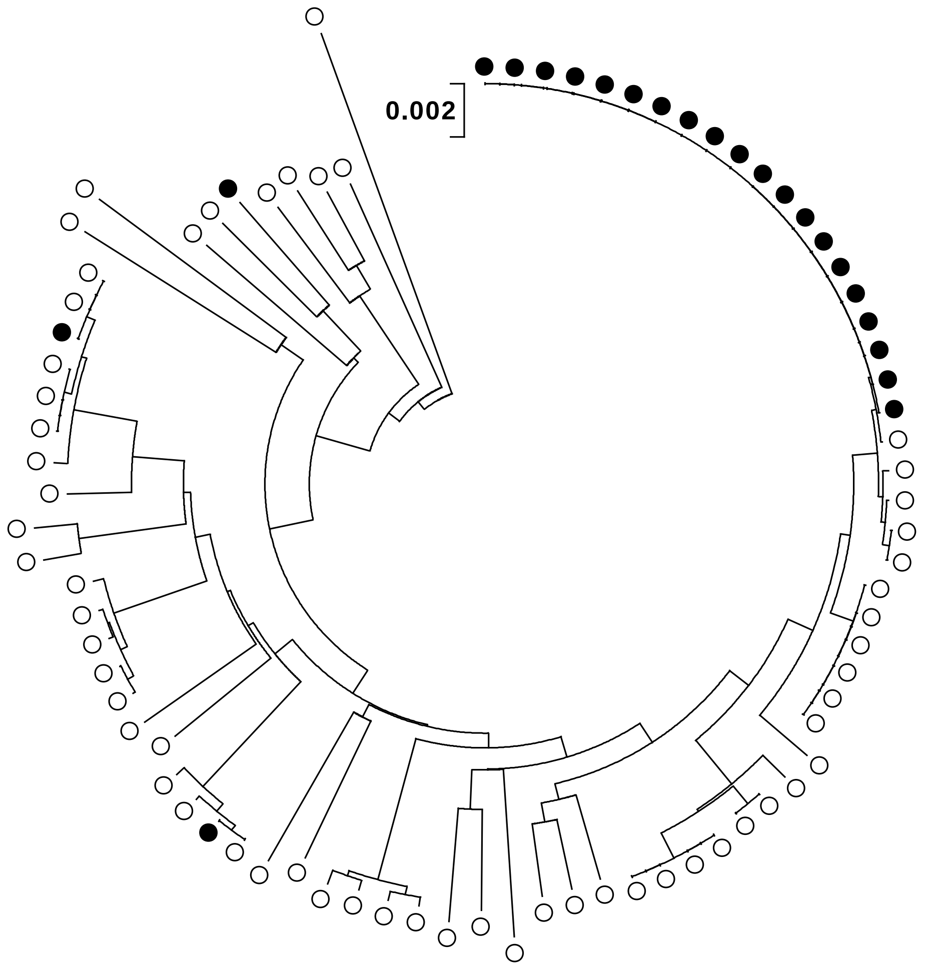

Figure S1. Identification of outbreak isolates from a contemporaneous population of clinical samples using cgMLST. Nucleotide sequence of cgMLST alleles from 23 potential outbreak isolates from an isolated population, and 59 contemporaneous *C. jejuni* isolates from Oxfordshire, UK, were used to reconstruct a Neighbour Joining tree. Potential outbreak isolates are represented as solid black circles and contemporaneous *C. jejuni* as black rings. The scale bar represents p-distances between aligned sequences.
